# Coin-sized, fully integrated, and minimally invasive continuous glucose monitoring system based on organic electrochemical transistors

**DOI:** 10.1126/sciadv.adl1856

**Published:** 2024-04-19

**Authors:** Jing Bai, Dingyao Liu, Xinyu Tian, Yan Wang, Binbin Cui, Yilin Yang, Shilei Dai, Wensheng Lin, Jixiang Zhu, Jinqiang Wang, Aimin Xu, Zhen Gu, Shiming Zhang

**Affiliations:** ^1^ Department of Electrical and Electronic Engineering, The University of Hong Kong, Pokfulam Road, Hong Kong SAR, China.; ^2^ School of Biomedical Engineering, Guangzhou Medical University, Guangzhou, China.; ^3^ State Key Laboratory of Advanced Drug Delivery Systems, Key Laboratory of Advanced Drug Delivery Systems of Zhejiang Province, College of Pharmaceutical Sciences, Zhejiang University, Hangzhou, China.; ^4^ State Key Laboratory of Pharmaceutical Biotechnology, The University of Hong Kong, Pokfulam Road, Hong Kong SAR, China.; ^5^ Jinhua Institute of Zhejiang University, Jinhua, China.

## Abstract

Continuous glucose monitoring systems (CGMs) are critical toward closed-loop diabetes management. The field’s progress urges next-generation CGMs with enhanced antinoise ability, reliability, and wearability. Here, we propose a coin-sized, fully integrated, and wearable CGM, achieved by holistically synergizing state-of-the-art interdisciplinary technologies of biosensors, minimally invasive tools, and hydrogels. The proposed CGM consists of three major parts: (i) an emerging biochemical signal amplifier, the organic electrochemical transistor (OECT), improving the signal-to-noise ratio (SNR) beyond traditional electrochemical sensors; (ii) a microneedle array to facilitate subcutaneous glucose sampling with minimized pain; and (iii) a soft hydrogel to stabilize the skin-device interface. Compared to conventional CGMs, the OECT-CGM offers a high antinoise ability, tunable sensitivity and resolution, and comfort wearability, enabling personalized glucose sensing for future precision diabetes health care. Last, we discuss how OECT technology can help push the limit of detection of current wearable electrochemical biosensors, especially when operating in complicated conditions.

## Teaser

An advanced CGMs synergizes cutting-edge biosensors, minimally invasive tools, and hydrogels toward precision diabetes management.

## INTRODUCTION

Diabetes mellitus is one of the most malignant chronic diseases threatening human health (*1*, *2*). Improper interventions for patients with diabetes mellitus may cause sudden hypoglycemia and increase the risk of complications (*3*, *4*), emphasizing the importance of accurate detection of blood glucose levels. The past two decades have witnessed intensive progress in continuous glucose monitoring systems (CGMs) (*5*–*8*). CGMs can inform, notify, and alert patients with diabetes of sustained hyperglycemia and incident hypoglycemia and are indispensable devices to enable closed-loop blood glucose control systems (*9*, *10*). Furthermore, CGMs have been recently adopted by healthy individuals to modify their dietary habits, aiming to achieve wellness and weight loss goals, demonstrating a technology-enabled new lifestyle (*11*).

Despite its importance, existing wearable CGM devices still face issues, including pain during sensor implantation, which deters patient usage (*12*–*14*). An emerging solution is to develop fully integrated and minimally invasive technologies to mitigate the pain. A promising technology is to use microneedles with a length of around 1 mm to detect interstitial fluid (ISF) glucose (*13*–*15*). Two general approaches can be used to integrate the glucose sensors with a microneedle, yet both present challenges. One is to directly fabricate the sensor onto the tiny needle, but it poses challenges on microfabrication (*16*). The second is to bridge the sensor with a hollow microneedle through a diffusive buffer layer, where a controlled diffusion and good interface stability are crucial, especially under motion (*13*, *14*).

In addition to the above issues, sensors with new features are being pursued for future precision diabetes health care (*17*–*19*). In this context, organic electrochemical transistors (OECTs) stand out because of their ability to synergize electrochemistry and transistor amplifiers for a better sensing quality (*20*–*28*). OECTs based on organic mixed ion-electron conductors (OMIEC), e.g., poly(3,4-ethylenedioxythiophene):poly (styrene-sulfonate) (PEDOT:PSS), can operate in aqueous environments at low voltage (<1 V) with low-power consumption while maintaining stable performance over months (*29*–*37*). The operation of OECTs relies on the electrochemical doping/de-doping process of the OMIEC semiconducting channel in contact with the electrolyte, where mobile ions can move freely to modulate the whole channel bulk (*36*, *38*–*43*). These properties endow OECTs with a record-high amplification ability (quantified as transconductance, *G*_m_), enhancing the signal-to-noise ratio (SNR) (*44*), making them ideal technologies for detecting weak biosignals in vivo (*44*–*47*), simultaneously addressing the inherent low-power/high-gain tradeoff in biomedical sensors (*48*).

To date, studies on OECT-based glucose biosensors have mostly concentrated on enhancing sensors’ amplification ability (i.e., *G*_m_), mechanical stretchability, and operational stability (*25*, *49*, *50*). Toward those goals, Zhu *et al.* (*51*) reported an OECT-based glucose sensor working in a neutral pH environment. Tang *et al.* (*52*) demonstrated a highly selective glucose sensor by modifying the gate electrode of the OECTs with carbon nanotubes. Macaya *et al.* (*53*) proposed an OECT-based glucose sensor with micromolar sensitivity where the gate electrode was coated with a gel containing glucose oxidase (GOx). Li *et al.* (*50*) reported stretchable OECTs for glucose detection, where the device remains functional under up to 30% of strain. Most recently, Wang *et al.* (*54*) reported a high-*G*_m_ OECT demonstrating sensitive detection of glucose with a *G*_m_ of 180 mS.

Although OECT glucose biosensors have undergone extensive study, their practical application as a CGM in a real wearable scenario remains unexplored due to a lack of system-level development strategies (*55*). Moreover, to ensure their competitiveness as next-generation CGMs, OECT glucose sensors must be integrated with miniaturized readout systems for comfort wearability (*27*, *56*) and minimally invasive sampling technologies to reduce discomfort during skin penetration (*15*, *57*). Furthermore, strategies that can ensure the stability of skin-device interfacing are an indispensable component in completing the system puzzle (*58*, *59*).

Here, we present a wearable CGM based on OECT technology (OECT-CGM), customized for next-generation precision diabetes health care. This compact, coin-sized, fully integrated, wireless OECT-CGM system encompasses the following key elements: (i) an OECT serving as the glucose biosensor for on-site signal amplification; (ii) a microneedle array serving as the minimally invasive bridge for ISF sampling; and (iii) a robust, adhesive, and enzyme-loaded hydrogel to improve the skin-device interface and enhance sensing reliability. These components were packed into a coin-sized entity, facilitated by the integration with the Personalized Electronic Reader for Electrochemical Transistors (PERfECT) (*56*). Compared to conventional CGM systems based on electrochemical sensing technology, OECT-CGM can offer improved antinoise ability and on-demand tunable sensitivity and resolution, which are critical for wearable applications. A self-calibration method is propsosed to help assess the real-time condition of the OECT sensor. Last, we demonstrated the viability of the OECT-CGM system for monitoring glucose levels, both in vitro and in vivo.

## RESULTS

### Design principle of the OECT-CGM system

**Fig. 1.**
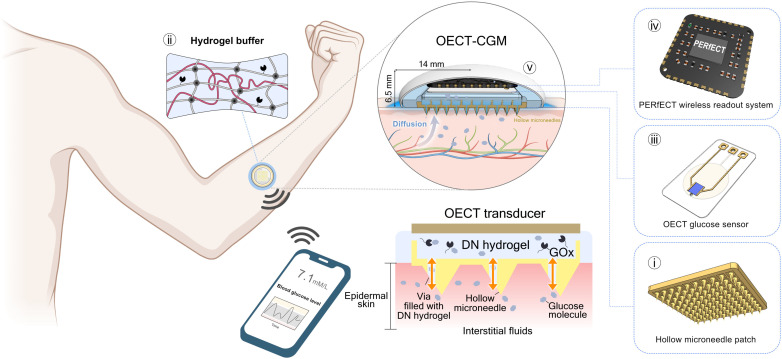
The concept and design principle of the OECT-CGM system. The OECT-CGM consists of: (**i**) a microneedle patch, (**ii**) a soft and adhesive hydrogel buffer membrane, (**iii**) an OECT glucose sensor, (**iv**) a miniaturized OECT readout system (PERfECT), and (**v**) a 3D-printed resin encapsulation case. The integration was strategically optimized to minimize the overall size of the system for a comfort wearability.

The OECT-CGM system (Fig. 1 and fig. S1) comprises the following units: (i) a hollow microneedle patch, (ii) a soft, adhesive, and enzyme-loaded hydrogel membrane, (iii) an OECT-based glucose sensor, (iv) a miniaturized readout system (PERfECT) (*56*), and (v) a three-dimensional (3D)–printed resin encapsulation layer. The microneedle serves as a minimally invasive bridge between the ISF and the OECT sensor. The adhesive enzyme-loaded hydrogel membrane is sandwiched between the microneedle patch and the OECT glucose sensor to enhance interfacing stability during motion. The glucose molecules in ISF passively diffuse to the OECT biosensor (through the microneedle and hydrogel) driven by the concentration gradient (Fig. 1) (*13*, *14*, *60*). The hydrogel membrane is synthesized by constructing an interpenetrating network (IPN) structure of polyacrylamide (PAAm) and Na^+^-alginate (*61*). This double-network (DN) hydrogel was further loaded with GOx for glucose detection. The OECT current is recorded by the PERfECT system, which can communicate with mobile phones. We used a foldable flexible printed circuit board (fPCB) connector as the power line, facilitating an easy connection between the OECT sensor and the PERfECT system. The above stacking strategy minimizes the physical dimensions (width and length) of the OECT-CGM system, optimizing its wearability.

### Fabrication and characterization of OECT glucose sensor

**Fig. 2.**
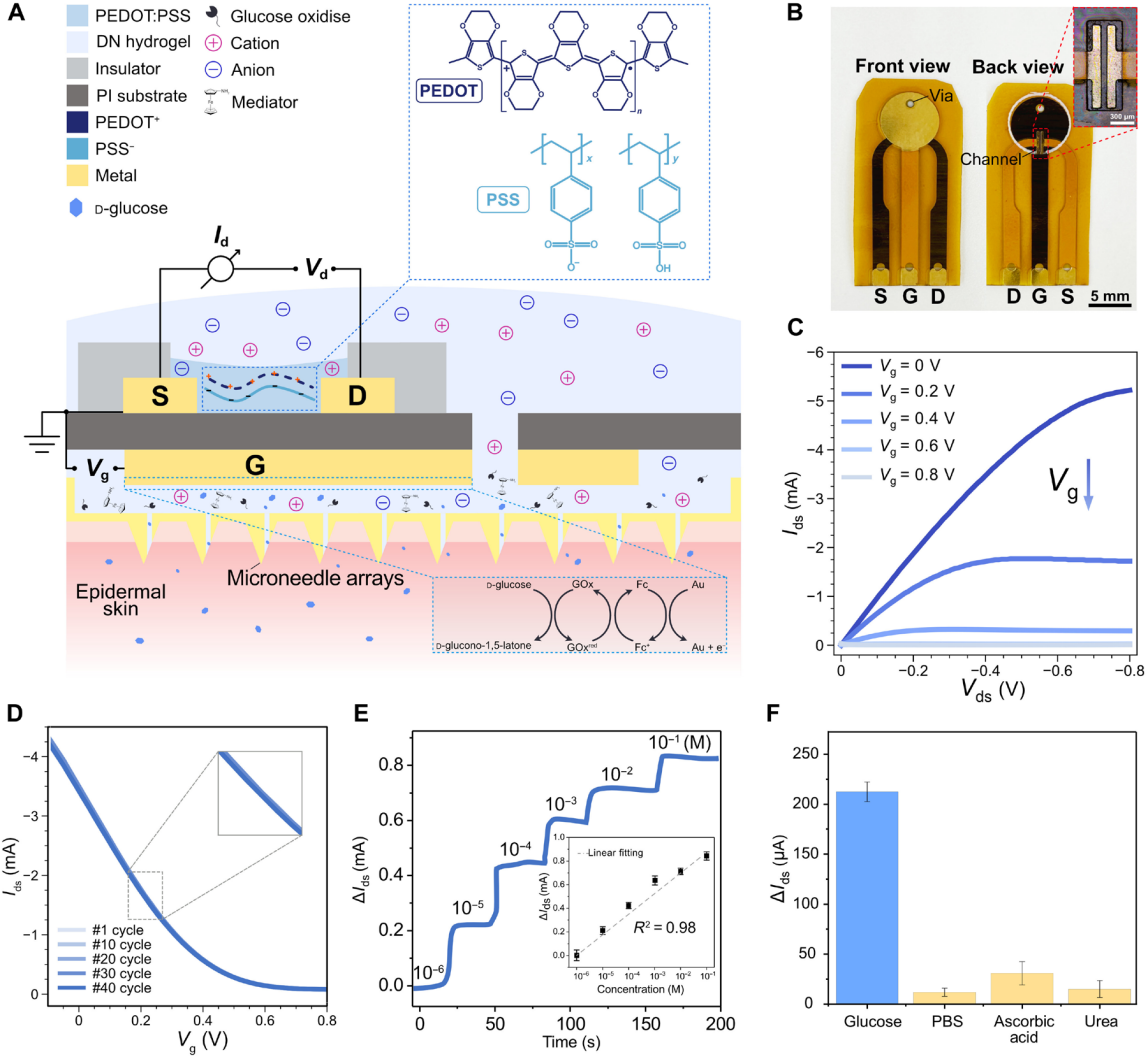
Fabrication and characterization of OECT-based glucose sensor. (**A**) Schematic of the OECT glucose sensor and the sensing mechanism. (**B**) The optical image of an OECT glucose sensor fabricated on a flexible substrate (PI). (**C**) Output curves of flexible OECTs, where *V*_ds_ were swept from 0 to 0.8 V with a step of 200 mV. (**D**) Transfer curves of the OECT with *V*_g_ swept from −0.1 to 0.8 V, with a step of 200 mV, repeating for 40 cycles. (**E**) Real-time current response (∆*I*_ds_) of OECT-based glucose sensor in response to glucose concentration ranging from 10^−6^ to 10^−1^ M (*n* = 5). Data are presented as means ± SD. (**F**) Response of OECT glucose sensors to potential interfering substances, including ascorbic acid and urea (*n* = 5). Data are presented as means ± SD. PBS, phosphate-buffered saline.

The OECT glucose sensor configuration is shown in Fig. 2 (A and B). We fabricated the OECT glucose sensor on a flexible polyimide (PI) substrate (detailed in Materials and Methods). A “back-to-back” design is implemented, positioning the gate electrode on the back and the channel on the front of the substrate. This back-to-back assembly method avoids area competition and diminishes the risk of cross-contamination of the channel during bioreceptor modification on the gate. A via is drilled to connect the front and back sides of the substrate to allow ion transport between the gate and the channel. (Fig. 2B). The OECT showed typical transistor output curves working in depletion mode (Fig. 2C). The operational stability of the device was verified through cyclic transfer curves. A negligible shift was observed after the cyclic measurements (Fig. 2D), underscoring the sensor’s commendable stability for biosensing.

To assemble an OECT glucose sensor, GOx is loaded into a stretchable, IPN hydrogel membrane. The sensing mechanism is illustrated in Fig. 2A (*27*), which includes the following processes: (i) glucose oxidation: when glucose is presented, it is oxidized by the GOx embedded in the hydrogel, producing gluconolactone and reducing GOx; (ii) enzyme oxidation: the reduced form of GOx is oxidized by the mediator aminoferrocene and regenerated to the initial form. In turn, the aminoferrocene mediator becomes reduced; (iii) mediator oxidation: the reduced form of the aminoferrocene mediator is then oxidized back to aminoferrocene at the gate electrode surface, lastly relaying the electrons to the gate electrodes. Both GOx and aminoferrocene were covalently immobilized within the hydrogel to avoid leakage for long-term use (fig. S2). The sequential reactions mentioned above result in a faradic current (*I*_g_), associated with a minor change in gate voltage (Δ*V*_g_). This slight change (Δ*V*_g_) is then amplified by the OECT causing a larger change in *I*_ds_ (i.e., multiplying Δ*V*_g_ by a factor of *G*_m_) (fig. S3), finally enhancing the SNR.

The fabricated OECT biosensor demonstrated linear responses to a wide range of glucose concentrations, ranging from 10^−6^ to 10^−1^ M (Fig. 2E). Interference experiments showed the sensor with negligible responses to other substances, such as ascorbic acid and urea, thanks to the high selectivity of GOx (Fig. 2F). These findings indicate the suitability of the OECT glucose biosensor for analyzing bodily fluids in practical wearable scenarios.

### Design of a compact and wearable OECT readout system

**Fig. 3.**
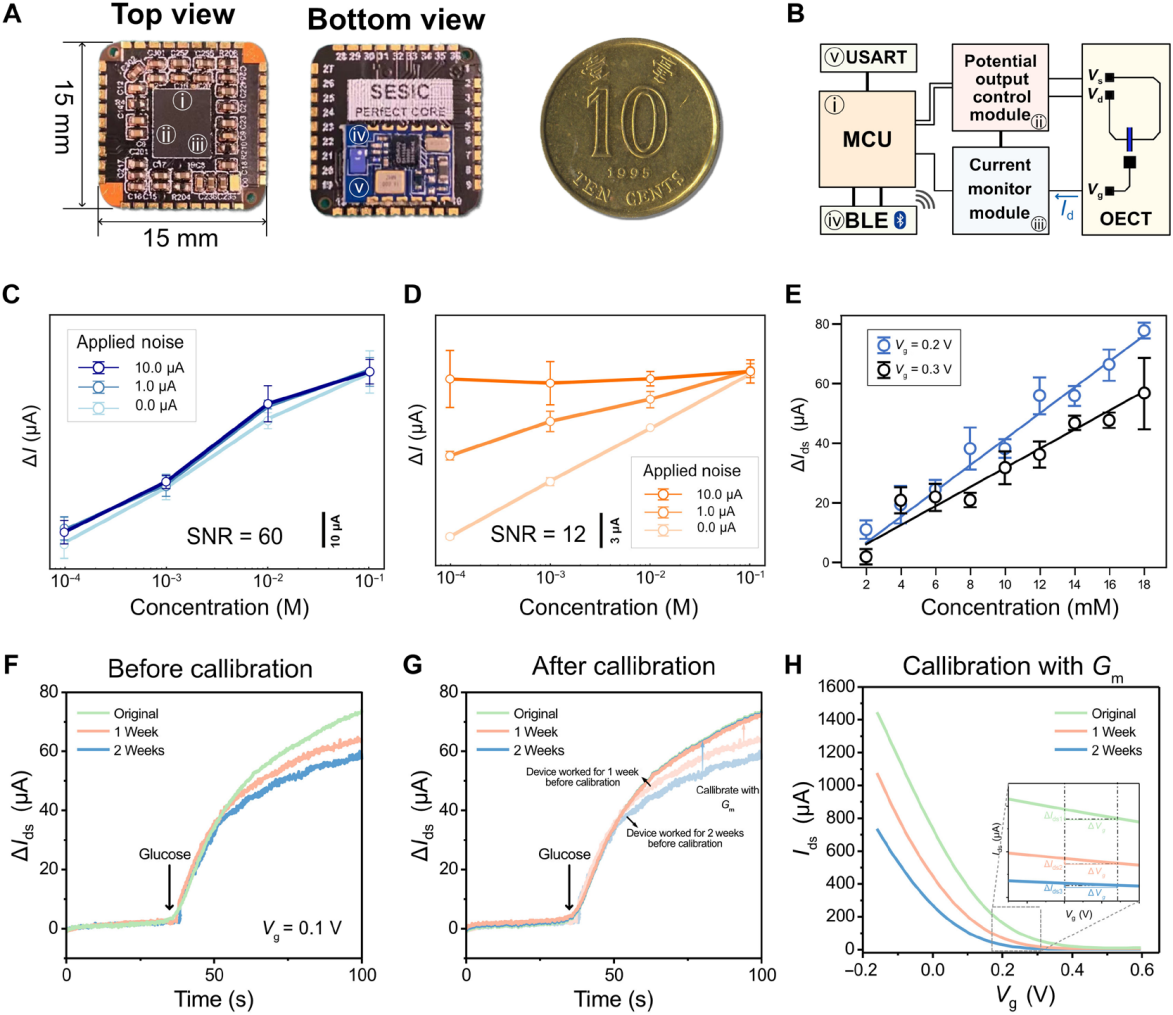
Integrated OECT glucose sensing platform with high antinoise ability, tunable sensitivity (resolution), and self-calibration ability. (**A**) Optical images of the PERfECT readout system customized for wearable OECT characterizations. (**B**) Circuit diagram of the PERfECT system. BLE, Bluetooth Low Energy; USART, Universal Synchronous Asynchronous Receiver and Transmitter. (**C**) Real-time current response (∆*I*_ds_) of OECT-based glucose sensor in response to glucose concentration changing from 10^−4^ to 10^−1^ M under different noise levels (*n* = 5). Data are presented as means ± SD. (**D**) Real-time current response (∆*I*, current of the working electrode) of an electrochemical glucose sensor in response to glucose concentration changing from 10^−4^ to 10^−1^ M under different noise levels (*n* = 5). Data are presented as means ± SD. Three white noises with different amplitudes (10.0, 1.0, and 0.2 μA) are used. The three different noises are superimposed on both electrochemical sensors and OECT sensors. The current response of the OECT-based sensor remains stable under different glucose concentrations. In contrast, the current response of the electrochemical sensor lost linearity under the noise of 10.0 μA. Data are presented as means ± SD. (**E**) Linear fitting of the current response of OECT to different glucose levels (2 to 18 mM), demonstrating a tunable sensitivity by controlling the *V*_g_ value. (**F**) The current response of OECT glucose sensors under prolonged usage up to 2 weeks (before calibration). (**G**) The calibrated current response of OECT glucose sensors. (**H**) The calibration process was performed by normalizing the *G*_m_, extracted from transfer curves measured at different stages.

A compact and lightweight readout system is an essential component for assembling a wearable sensor. Correspondingly, we developed PERfECT (*56*), a coin-sized readout system (dimension of 1.5 cm by 1.5 cm by 0.2 cm, weight of 0.4 g) that seamlessly integrates with smart wearables (Fig. 3, A and B). The logic diagram of the PERfECT system is shown in Fig. 3B. The potential control module takes commands from the microcontroller unit (MCU) to control the *V*_g_ and *V*_ds_ with steps down to 2 mV for precise characterization of the dynamic behaviors of the OECT sensor. The current monitor module can read the *I*_ds_ with the detection limit down to 1 nA, endowing PERfECT with benchmarkable resolution in both applying *V*_g_ and *V*_ds_ and reading *I*_ds_ when compared with the lab-used source measure unit (fig. S4).

### High antinoise ability, tunable sensitivity, and self-calibration of OECT glucose sensor

In contrast to traditional electrochemical sensors, the proposed OECT glucose sensors have an inherently higher current (*I*_ds_) (Figs. 2E and 3, C and D). They demonstrated higher Δ*I*_ds_ in response to glucose changes, achieved through the integration and amplification of the gate signal (*I*_g_) (fig. S5) (*62*). This high Δ*I*_ds_ makes the sensor resilient to environmental noises, a desired feature in challenging wearable scenarios. Figure 3C and fig. S6 confirm that OECT sensor owns a higher SNR compared to a traditional electrochemical sensor (~60 dB versus ~10 dB). The high SNR renders OECTs to maintain a stable linear detection curve in a simulated noisy environment (Fig. 3C), whereas the electrochemical sensor loses sensitivity under the same conditions (Fig. 3D and fig. S7).

Tunable sensitivity and adjustable resolution are sought in biosensors (*63*), particularly useful when we need to focus on a weak signal change within a specific range of interest. In an integrated biosensing system, signal transmission and processing are digitalized and discrete. The smallest unit of a digital signal linearly corresponds to the minimum distinguishable unit of glucose concentration, with the slope representing the sensitivity. Therefore, adjusting sensitivity (slope) allows fine-tuning the resolution on demand. Achieving high resolution is crucial for accurately predicting glucose levels and enabling future precise diabetes health care.

As shown in Fig. 3E, tuning the *V*_g_ from 0.3 to 0.2 V increased the sensitivity from 0.0035 to 0.0042 μA mM^−1^. The reason that the sensitivity can be tuned by *V*_g_ is because the *G*_m_ of OECTs, relevant to the sensitivity, has a linear relationship with *V*_g_, governed by the following Eq. 1.

$$G_{m}=\frac{Wd}{L}\mu C^{*}\left(V_{\text{th}}-V_{g}\right)$$ (1)

where *W* is the channel width, *d* is the channel thickness, *L* is the channel length, μ is the charge carrier mobility, C* is the volumetric capacitance, *V*_th_ is the threshold voltage, and *V*_g_ is the gate voltage (*64*).

While *G*_m_ brings OECTs a high sensitivity, its variation can result in deteriorated sensing, especially during prolonged use. Therefore, calibration of OECTs is needed to assess their condition before each use. The PERfECT readout system allows performing the self-calibration of OECTs by first examining the condition of the enzyme-loaded gate electrode with impedance analysis (fig. S8), followed by reading the *G*_m_ of OECTs (Fig. 3, F to H, and fig. S9). As shown in Fig. 3F, the sensor’s current response (Δ*I*_ds_) to glucose decreases within 2 weeks due to the decrease of *G*_m_. After calibration by normalizing the *G*_m_, the Δ*I*_ds_ become identical (Fig. 3G), allowing the reset of the sensor during the usage period (Fig. 3H).

### Prototyping of the OECT-CGM system

#### Microneedle for minimally invasive ISF sampling

**Fig. 4.**
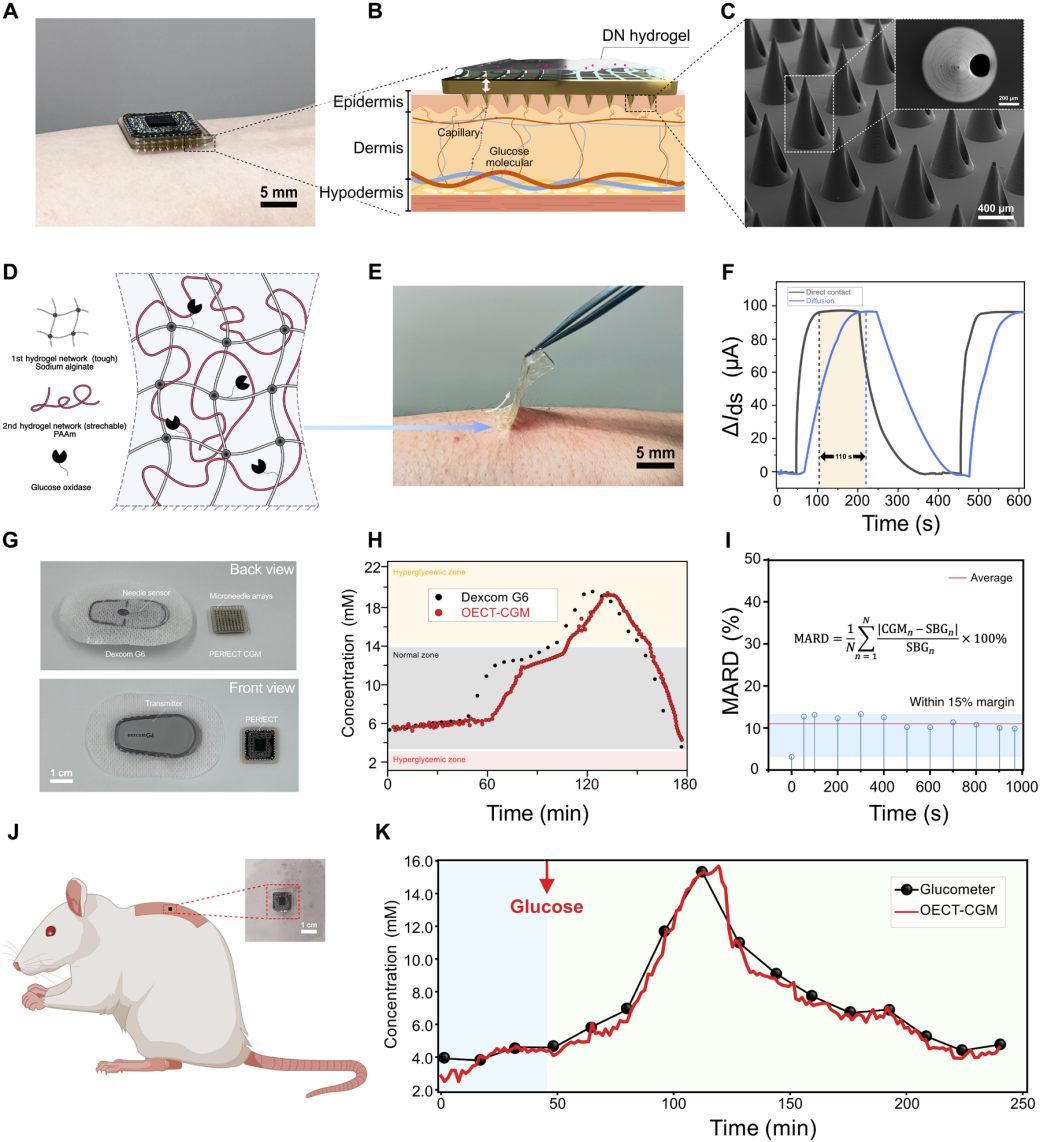
Prototyping of the fully integrated OECT-CGM and the in vivo evaluation. (**A** to **C**) Optical image of the OECT-CGM placed on the skin (A); glucose diffusion mechanics between ISF and the OECT via the microneedle (B); and scanning electron microscopy image of the microneedle (C). (**D**) Illustration of the adopted adhesive and soft hydrogel. (**E**) Optical images of the adhesive and soft hydrogel during peeling off. (**F**) Delay time of sensor response with hydrogel serving as a diffusive layer. (**G**) Comparison of the size of the OECT-CGM with Dexcom G6. (**H**) Comparison of real-time recording results between OECT-CGM and Dexcom G6 (in vitro). (**I**) The MARD value of OECT-CGM (calculated with 12 sampling points within 1000 s). (**J**) Schematic of in vivo test with a rat model. (**K**) Comparison of real-time recording results between OECT-CGM and Dexcom G6 (in vivo) (black, 16 data points of the glucose concentration collected by a glucometer; red, readout of glucose concentrations collected by OECT-CGM).

One of the main obstacles against the wide adoption of the current CGM system is the pain associated with the implantation process (*12*). Microneedles, with shortened needle lengths (<1 mm), have emerged as promising tools to enable minimally invasive sensing in the ISF rather than blood, thereby avoiding contact with neurons and reducing pain (Fig. 4, A and B) (*65*–*67*). To assemble the OECT-CGM system, we married the OECT glucose sensor with microneedles. To ease the skin penetration, we used a sharpened tapered shape of microneedle in 10 × 10 arrays, with a taper angle of 15°. The efficiency of this structure in penetrating the skin has been previously validated (Fig. 4C) (*13*, *14*). The size of the microneedle array is designed as 10 mm by 10 mm by 2.2 mm to be compatible with the CGM readout system (fig. S10). To improve the biocompatibility of the microneedle, we metalized the surface of the microneedle by depositing a thin layer of Au (~100 nm) (figs. S11 and S12 and Materials and Methods).

#### Hydrogel adhesive improves the skin-device interfacing

Robust adhesion between the sensor and skin is a crucial factor in improving the durability and wearability of CGMs (*27*). Toward this goal, we introduced a soft and adhesive buffer layer by hybridizing an IPN hydrogel with a bioadhesive elastomer (detailed in Materials and Methods). The IPN hydrogel consists of a tough Na^+^-alginate hydrogel (primary network) and a soft PAAm hydrogel (secondary network) (*61*). The hydrogel may be further mixed with GOx and mediator to serve as a solid-state glucose-sensing membrane to simplify the fabrication of glucose-sensing electrodes for a CGM (Fig. 4, D and E) (*68*, *69*). The soft hydrogel and the adhesive elastomer ensure the robustness of the skin-device interface under motions (movie S1) (*70*, *71*).

#### Hydrogel-assisted glucose diffusion from ISF to OECT sensor

Compared to implantable sensors, skin-attachable ones are preferred for simplified device assembly and longer lifetime by minimizing foreign body reactions (*13*, *14*). The skin-attachable sensor works by taking advantage of the gradient-induced passive glucose diffusion from the ISF to the sensor (*13*, *14*). Here, we used a porous hydrogel to facilitate the glucose diffusion (Fig. 4B and figs. S13 to S16) while avoiding potential clogging issues at the tips of the microneedles. Citrate ions were added into the hydrogel to suppress the healing process (*72*).

Glucose diffusion dynamics in the hydrogel play a key role in determining the sensing performance. To quantify the diffusion, we measured the glucose diffusion coefficient with electrochemical impedance spectroscopy (*73*–*76*), by analyzing the Warburg coefficient (σ) which models semi-infinite linear diffusion (fig. S15). The σ is expressed as follows which depends on multiple factors (*77*)

$$\sigma =\frac{\text{RT}}{n^{2}F^{2}A\sqrt{2}}\left(\frac{1}{D_{O}^{1/2}C_{O}^{*}}+\frac{1}{D_{R}^{1/2}C_{R}^{*}}\right)$$ (2)

where *n* is the number of electrons transferred, *R* is the gas constant, *T* is the temperature, *F* is the Faraday’s constant, *A* is the electrode surface area, *D*_O_ is the diffusion coefficient of the oxidized species (i.e., glucose), C*_O_ is the bulk concentration of the oxidized species (glucose), *D*_R_ is the diffusion coefficient of the reduced species, and C*_R_ is the bulk concentration of the reduced species. By using the hydrogel-coated working electrode, we obtained a *D*_O_ of 3.90 × 10^−12^ cm · s^−1^ for glucose, close to the *D*_O_ in water (6.54 × 10^−12^ cm · s^−1^), thanks to the high porosity and hydrophilicity of the hydrogel. Nevertheless, despite its effectiveness, a lag of approximately 120 s was observed compared to the reference sensor in direct contact with the ISF. The lag was consistent regardless of glucose concentrations (1 to 10 mM), in line with other microneedle-based sensors which can be attributed to the delayed glucose diffusion from the ISF to the OECT sensor (Fig. 4F) (*13*, *14*).

#### In vitro validation of the OECT-CGM

To validate the OECT-CGM system, we compared it with a reference CGM system (Dexcom G6) (Fig. 4G and fig. S17) (*6*). The reference device consists of a needle-based sensor with a length of ~2.0 cm, an adhesive patch, a supporting frame, and a wireless data transmitter. The OECT-CGM system consists of a microneedle array with a needle length of 1.0 mm, an adhesive layer, an OECT glucose sensor, and the PERfECT readout system. An artificial skin, penetrated by the microneedle, was used to replicate the skin-device interface. During the in vitro experiments, we dynamically controlled the glucose levels in the chamber with a pump and monitored the values with both the OECT-CGM system and the reference device. During the 180-min test, the concentration was varied between 3 and 20 mM. As shown in Fig. 4H, the OECT-CGM showed comparable results with the reference device but with a smaller size (1.5 cm by 1.5 cm) and weight (Fig. 4G). A response delay of ~8 min was observed in the OECT-CGM system, attributable to the diffusion delay of glucose molecules in the hydrogel. The OECT-CGM exhibited a mean absolute relative difference (MARD) value of ~15%, demonstrating its potential for future practical use (Fig. 4I).

#### In vivo validation of the OECT-CGM

Last, we validated the OECT-CGM in vivo with rats, as depicted in Fig. 4J and fig. S18. To induce hyperglycemic conditions, we administered glucose solutions to manipulate the rats’ blood glucose levels. After reaching the peak, the blood glucose levels automatically returned to normal ranges. The rats exhibited a peak blood glucose level of ~16 mM approximately 1 hour after the administration of the glucose solution. We monitored glucose levels over a 250-min period, and the results were compared with the reference device (Fig. 4K). Despite minor fluctuations, the trend from both sensors showed good consistency within the detection range from 2 to 16 mM, demonstrating the effectiveness and reliability of OECT-CGM for in vivo applications.

## DISCUSSION

In conclusion, we present a coin-sized, fully integrated wearable OECT-CGM. This system synergistically combines OECT biosensors, microneedles, and diffusive hydrogels, all separately optimized for seamless integration and customizable biosensing. We conducted both in vitro and in vivo experiments to assess the viability of the OECT-CGM for future personalized and precision diabetes health care.

**Fig. 5.**
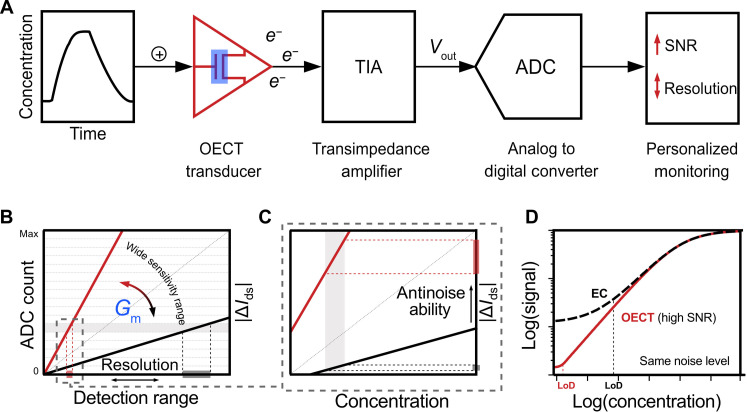
Illustration of holistic control of SNR, LoD, sensitivity, and resolution by using OECT as biosignal amplifier in an integrated biosensing system. This can be achieved through wide-tuning the *G*_m_, making it competitive for customizable wearable health care applications. (**A**) The diagram illustrates the entire sensing process in an integrated sensor system, from the measured analog signal to the terminal digital signal. The measured signal (Δ*V*) is first amplified in situ by the OECT (*G*_m_ × Δ*V*) and then further amplified by the transimpedance amplifier (TIA) (with a resistor, *R*_TIA_) and converted into a voltage signal (*G*_m_ × Δ*V* × *R*_TIA_). Subsequently, it is converted into a digital signal through the analog-to-digital converter (ADC). By using the front-end deployed OECT and its *G*_m_ value, we can avoid amplifying the online noise, enabling a more efficient regulation of the SNR and resolution of the digitalized signal. (**B**) Illustration of how the sensor’s resolution is controlled by adjusting *G*_m_ values. The ADC counts (*y* axis) and the detection range (*x* axis) are correlated. As *G*_m_ increases, limited by the ADC counts, the detection range narrowed to lower concentrations, achieving a higher resolution (from gray to red, highlighted in a dashed box on the *x* axis). (**C**) Simultaneously, the current change caused by a unit signal increases (from gray to red, highlighted in a dashed box on the *y* axis, indicated as |Δ*I*_ds_|), enhancing antinoise ability. (**D**) Therefore, increasing the *G*_m_ not only increases the sensitivity but also improves the antinoise ability (thus SNR). The synergy of these two co-enhanced factors can help push the LoD of an electrochemical (EC) biosensor (indicated as the red curve). This is highly desired in complex wearable scenarios where the noise level can be much higher than in common cases.

Compared to conventional electrochemical biosensor (*6*, *7*), the OECT biosensor can offer the following additional features (Fig. 5, A to D): (i) enhanced SNR achieved by leveraging its high *G*_m_ and on-site amplification potential (Fig. 5B), thus enabling lower limit of detection (LoD) [discussed in detail in Fig. 5 (B to D)]; (ii) tunable sensitivity and resolution achieved by tuning *G*_m_ over a wide range, providing an opportunity to balance between detection range and signal resolution (Fig. 5A); both critical for practical applications in diverse wearable scenarios. Moreover, these two essential parameters can be adjusted by the OECT transducer itself, which simplifies peripheral circuit design during sensor integration.

Compared to other transistor-based transducers, OECTs stand out due to their ability to simultaneously combine the following features: (i) high *G*_m_ owing to the unique bulk modulation mechanism (*20*); (ii) high channel current due to the high conductivity of PEDOT:PSS, making it immune to environmental turbulence; (iii) scalability allowing fabrication of large-sized devices (up to millimeter scale) (*27*, *78*) with cost-effective fabrication methods while maintaining a high *G*_m_; and (iv) suitability for in vivo applications due to the high stability (based on PEDOT:PSS), lower operation voltage (<1 V), and tunable mechanical properties (*47*). Both flexible and stretchable OECTs (*58*, *59*, *79*, *80*) have been developed to improve the skin-device interface, but such properties remain challenges for the majority of other transistor-based transducers due to limited material solutions and fabrication methods (fig. S19).

Associated with the aforementioned advantages, some limitations remain. OECT uses a PEDOT:PSS channel to amplify signals; concurrently, the stability of the channel must be ensured to maintain the sensor’s overall reliability. While previous works have reported the degradation of the OECTs (*81*), recent breakthroughs have demonstrated improved robustness of PEDOT:PSS thin films, maintaining good stability during 800 days of in vivo testing (*33*).

Another concern may exist in certain scenarios where the signal varies substantially, causing a considerable *V*_g_ change and, consequently, a *G*_m_ change. However, in most cases, this is not expected to be a major issue as OECTs are supposed to be used for detecting weak signals beyond electrochemical sensors’ detection range (Fig. 5B) (*82*, *83*). Because of the small signal variation, both Δ*V*_g_ and Δ*G*_m_ should be negligible, thus maintaining a linear response curve within the current window of interest. The current window can be predesigned holistically in an integrated biosensing circuit by tailoring the analog-to-digital converter and the *R*_transimpedance amplifier_ (Fig. 5A). Here, we discuss OECT properties by putting it into an integrated biosensing circuit rather than treating it as a separated transducer, which is crucial for practical applications.

The last concern is the frequency response. Despite not being comparable with silicon transistors, a megahertz response has been reported through device architecture design (*24*, *84*), far exceeding the required frequency to detect sluggish bioelectrochemical processes. We expect the presented work, together with the above discussions, can facilitate the use of OECTs in an integrated biosensing system for personalized and prolonged wearable CGM applications and beyond.

## MATERIALS AND METHODS

### Materials

PEDOT:PSS aqueous suspension (Clevios PH1000) was purchased from Heraeus Electronic Material. Glycerol, dodecylbenzene sulfonic acid (DBSA), sodium chloride, (3-glycidyloxypropyl) trimethoxysilane (GOPS), acrylamide (AAm), calcium chloride (CaCl_2_), and 3-(trimethoxysilyl) propyl methacrylate (TMSPMA) were purchased from the Sigma-Aldrich (USA). Hydroxy-4′-(2-hydroxyethoxy)-2-methylpropiophenone (Irgacure 2959), glutaraldehyde, aminoferrocene, GOx, and chitosan were provided by Aladdin Co. (Shanghai, China). The PI thin film was obtained from the DuPont Co. (USA). The biocompatible adhesive polyurethane was provided by 3M (USA). Unless otherwise specified, the chemicals in the current work were used without further purification.

### Animals

The animal work was approved by the Animal Ethics Committee of Guangzhou Medical University and conducted in accordance with relevant guidelines. Female Sprague Dawley rats (300 to 350 g) were purchased from the Guangdong Medical Laboratory Animal Center and acclimatized in an approved animal facility.

### Microneedle fabrication

To fabricate the hollow microneedle patch, a high-resolution 3D printer (S240 from Boston Micro Fabrication) is used. A minimal needle tip size of 15 μm was obtained by ultra-precision stereolithography technology of the printer. The microneedle structures were designed using computer-aided design software and then printed using a photocurable resin. The printed structures were then postprocessed by curing them in an ultraviolet (UV) light chamber and rinsing them with isopropyl alcohol to remove uncured resin. After the postcuring process, the microneedle patch surface was then metalized with Au by magnetron sputtering to enhance its strength and durability.

### OECT sensor fabrication

To fabricate the OECT on a flexible PI substrate, we started from constructing the source/drain/gate electrodes by depositing a layer of gold thin film, the pattern of which was defined by a shadow mask. Then, the active channel layer, PEDOT:PSS, was deposited between the source and drain electrodes by inkjet printing with a minimal droplet size of 1 pl. The printable PEDOT:PSS ink was prepared by firstly stirring for 3 min and then mixed with GOPS (1%, w/w), glycerol (5%, v/v), and DBSA (0.1%, v/v) with a Vortex (MX-S). The addition of glycerol was to increase the film conductivity. DBSA was added to facilitate the wetting property of films on substrates. Before printing, the mixed suspension was filtered with a polytetrafluoroethylene membrane (aperture size of 0.45 μm) to remove aggregates, thus avoiding nozzle clogging. Next, the insulation layer of UV-curable resin was also deposited using the inkjet printing system (SWA3060, Yiwu Yangtian Electronic Technology Co. Ltd., Yiwu, China) and cured in situ using the UV lamp equipped with the printer. To complete the OECT structure, a soft IPN hydrogel composed of PAAm/Na^+^-alginate double-network was prepared to bridge the gate electrode and the channel.

To synthesize the soft IPN hydrogel, we dissolved 2% (w/w) Na^+^-alginate, 13% (w/w) AAm, 0.015% (w/w) MBAA, and 0.24% (w/w) Irgacure 2959 in deionized water. The mixture was then centrifuged at 2000 rpm to remove air bubbles. The PAAm network was formed by curing the solution in a UV chamber (364 nm, 10 W power) for 60 min. The second network was formed by cross-linking the Na^+^-alginate via immersing the previous gel in CaCl_2_ solution (1 M) for 24 hours to reach an equilibrium state.

For glucose detection, we prepared an enzymatic hydrogel by embedding GOx and amino-functionalized ferrocene within a robust IPN hydrogel. In this hydrogel, GOx selectively reacts with glucose, while aminoferrocene serves as the redox mediator. The preparation involved initially freeze-drying the IPN hydrogel for 48 hours to create a double-network scaffold. This scaffold was then swollen overnight in an aqueous solution containing GOx (500 U/ml), aminoferrocene (10 mM), and glutaraldehyde (0.3%, w/w). After removing the residues by washing with deionized water, the enzymatic soft hydrogel can be obtained.

To make the IPN hydrogel adhesive, we then hybrid the hydrogel with a bioadhesive elastomer thin film (provided from 3M). The elastomer thin film was perforated with a hole punch (200 μm in diameter) to facilitate the diffusion of glucose between ISF and the OECT sensor. The adhesive elastomer was firstly treated with TMSPMA solution [100 ml of deionized water, 10 μl of acetic acid with pH 3.5, and 2% (w/w) of TMSPMA] and then washed with ethanol and dried. Then, gel precursor was dropped on the TMSPMA grafted elastomer to avoid bubbles and cured by UV light for 60 min. The IPN hydrogel formation follows the same procedure as described above. The grafted TMSPMA tends to interconnect the IPN hydrogel and elastomer to enhance the overall adhesion to substrates.

### Miniaturized readout system fabrication and evaluations

To shrink the size of the readout system, we modified the recently developed miniaturized OECT readout system PERfECT to meet the specific requirements for CGM. The PERfECT system was developed with customized firmware (including data sampling, filtering, and device control) and hardware synergizing a customized MCU, an Analog MCU (ADuCM355), a BLE unit, and other necessary components, all soldered onto a printed circuit board using standard fabrication processes. The readout system was then connected to the OECT glucose sensor using an fPCB connector. The typical power consumption of the PERfECT system when sampling at a rate of 1 sample/s was measured to be 1 mW using Keithley B2902B. The performance of the modified PERfECT system was evaluated by conducting in vitro experiments to monitor the glucose concentration.

### In vitro experiments

We conducted in vitro experiments to simulate dynamic glucose variations in the human body. The testing setup comprised a glucose solution reservoir with both an inlet and outlet, a programmable syringe that allowed precise control of the injection speed, and a programmable injecting pump enabling us to modulate the glucose concentration in the reservoir (fig. S17). We performed two cycles of concentration change within the range of 0 to 20 mM and vice versa, with a speed of 0.3 mM/min. Each concentration change cycle was completed in approximately 2 hours, mimicking the potential fluctuations of glucose levels in the body.

### In vivo experiments

The in vivo test of the OECT-CGM for glucose tracking was performed using female SD rats (300 to 350 g). The rats were fasted for 20 hours but were provided with water to obtain a stable initial blood glucose level. First, for blood collection convenience, rats were anesthetized using 2.5% (v/v) isoflurane. To manipulate the rats’ blood glucose levels, a 400 mM glucose solution was administered to the rats via a syringe at a dosage of 10 ml/kg, starting 40 min after the commencement of the experiment. The OECT-CGM was sticked to the back of the rat, and the sampling rate was set to 10 s per sample. Every 90 s, 10 sets of data were packed and transmitted to the mobile wirelessly. To validate the blood glucose level of the rats, the blood taken from the tail vein of rats was tested by a glucometer (Yuwell Accusure 590, China) at the frequency of 15 min for 2 hours.
