# Supplementary material for: Coin-sized, fully integrated, and minimally invasive continuous glucose monitoring system based on organic electrochemical transistors
Source: Sci Adv. 2024 Apr 19;10(16):eadl1856. doi: 10.1126/sciadv.adl1856 (PMC11029813; doi:10.1126/sciadv.adl1856)
Supplement: Supplementary file 1 — Figs. S1 to S19 Legend for movie S1 [file sciadv.adl1856_sm.pdf]

Science Advances
AAAS

Supplementary Materials for

Coin-sized, fully integrated, and minimally invasive continuous glucose monitoring system based on organic electrochemical transistors

Jing Bai *et al.*

Corresponding author: Zhen Gu, guzhen@zju.edu.cn; Shiming Zhang, szhang@eee.hku.hk

Sci. Adv. **10**, eadl1856 (2024)
DOI: 10.1126/sciadv.adl1856

The PDF file includes:

Figs. S1 to S19
Legend for movie S1

Other Supplementary Material for this manuscript includes the following:

Movie S1

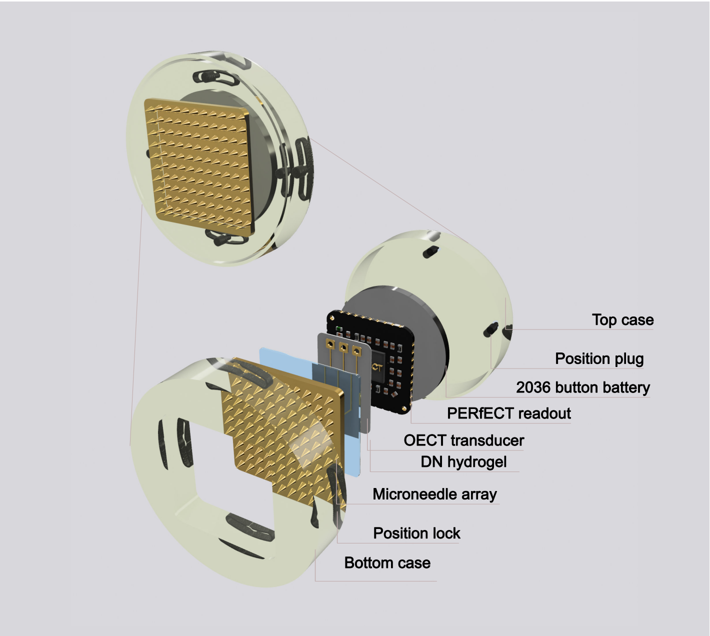

Fig. S1. Exploded view of the OECT-CGM system. The OECT-CGM system is assembled by the systematic integration and holistic synergy of four main components: i) a miniaturized personalized electronic reader for electrochemical transistors (PERfECT) (*57*); ii) an OECTs-based glucose sensor; iii) a hollow microneedle array; iv) a hydrogel layer to improve adhesion and robustness under deformation for stable glucose monitoring.

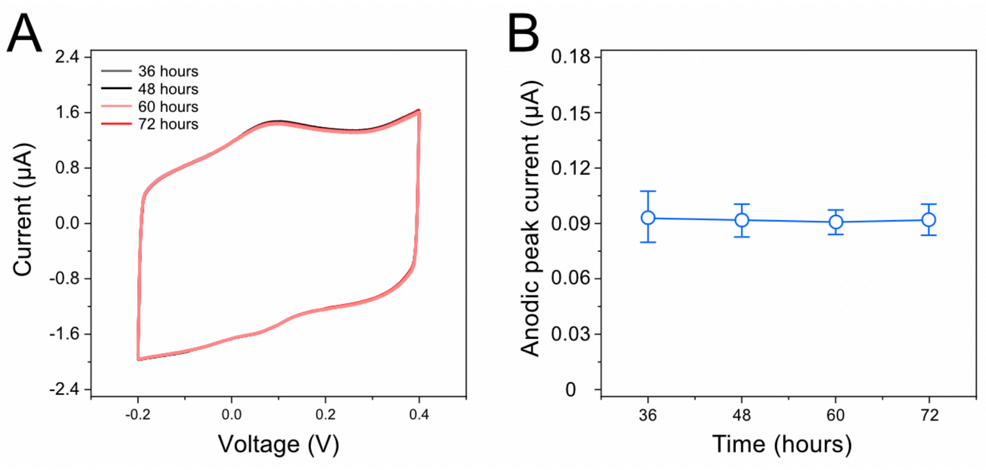

Fig. S2. Cyclic voltammetry (CV) curves for the enzymatic hydrogel over 72 hours. (A) The CV curves were obtained within a scanning range of -0.2V to 0.4V at a scan rate of 50 mV/s. **(B)** The minimal shift in the peak current over time indicates the effective retention of the amino ferrocene mediator within the hydrogel matrix.

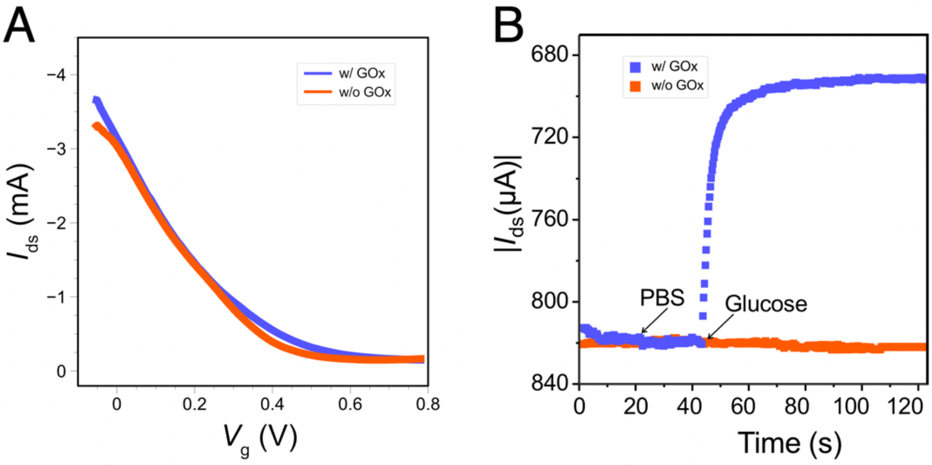

Fig. S3. Performance of OECT w/ and w/o GOx modification. (A) Transfer curves of OECT before and after GOx modification. **(B)** The sensor is first soaked in PBS solution After adding 2mL of 10 mM glucose solution to the test container at time = 46 s, the current of OECT with GOx decreased to 697 μA (V_g=0.2 V). In contrast, the OECT without GOx showed no response to glucose addition.

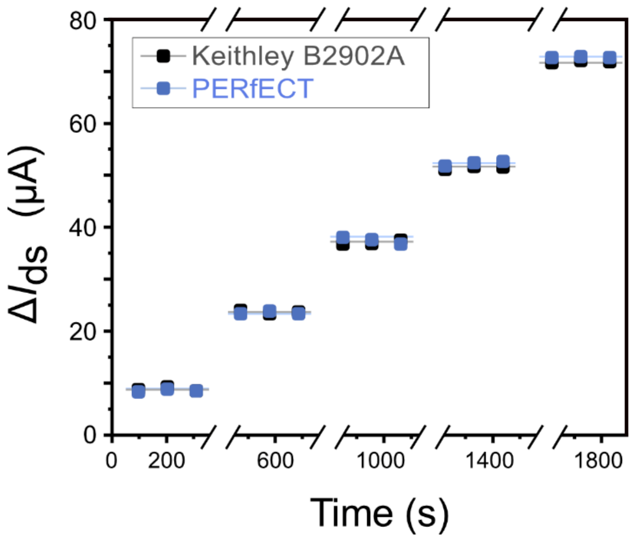

Fig. S4. Performance comparison of the PERfECT system and the lab-used SMU. Current responses of the OECT glucose sensor (2 mM to 18 mM, with a step of 4 mM) were measured by the PERfECT system (blue) and the lab-used SMU (black), indicating the high resolution of PERfECT system.

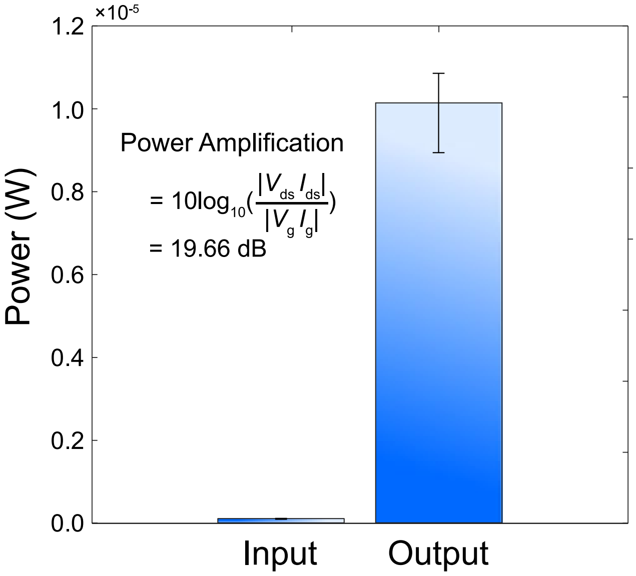

Fig. S5. OECT glucose sensor for signal amplification. The power amplification of biosignals in the OECT originates from the integration of I_g (*28*). The product of V_g and I_g represents the input power of the signal. The product of V_{ds} and I_{ds} represents the output power. The OECT glucose sensor demonstrated a power gain of 19.66 dB. n = 10. Data are means ± SD.

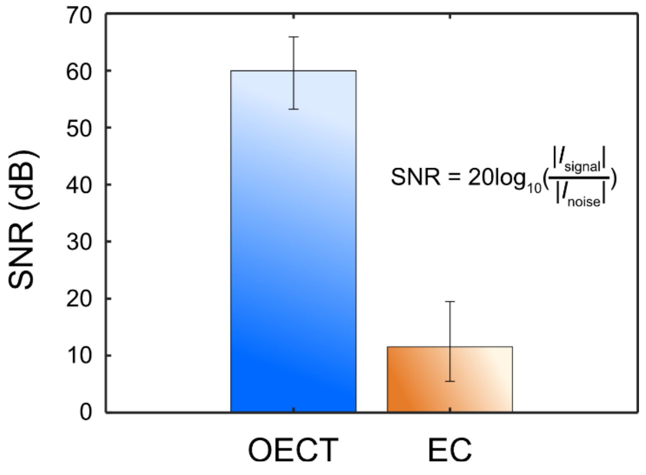

Fig. S6. SNR comparison of the OECT sensor and the EC sensor. SNR of both OECT and EC is calculated through the equation indicated in the inset of the figure. I_{signal} refers to the current of OECT or EC. I_{noise} refers to the simulated noise current. (n = 10). Data are presented as means ± SD.

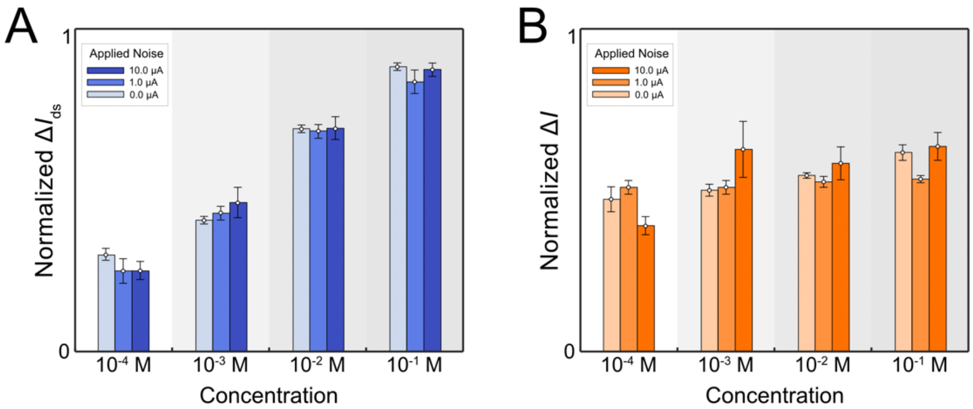

Fig. S7. Comparison of the anti-noise ability of OECT and EC glucose sensor. (A) The current response of the OECT glucose sensor under simulated noises of 0.0 μA, 1.0 μA, and 10.0 μA. The OECT-based sensor remained functional under 10.0 μA noise. **(B)** The current response of the EC glucose sensor under simulated noises of the amplitude of 0.0 μA, 1.0 μA, and 10.0 μA. The EC sensor lost function under 10.0 μA noise. (n = 5). Data are presented as means ± SD.

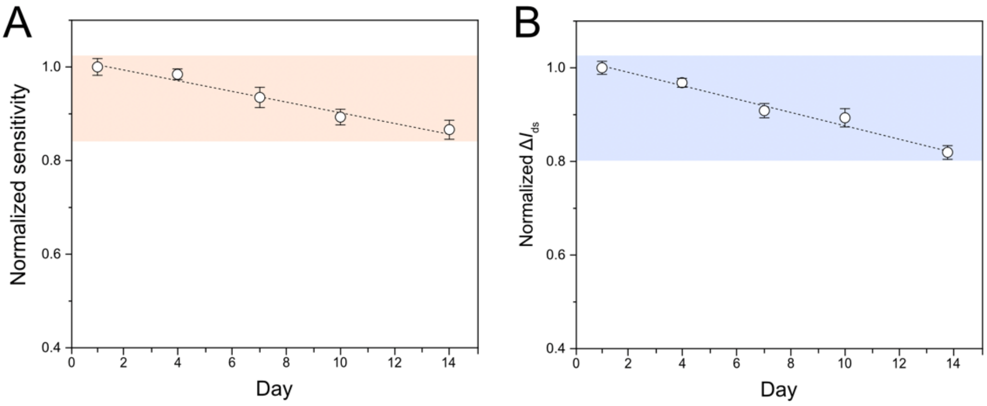

Fig. S8. In-lab assessment of the enzyme activity for 14 days. (A) Enzyme activity was evaluated, and the decay line was obtained *via* linear fitting. The enzyme activity showed a degradation of around 15% on Day 14 compared to the original. (n = 5). Data are means ± SD. **(B)** The stability (14 days) of an OECT glucose sensor. The decline in current indicates a decrease in the sensor's response to the same glucose concentration over the testing period. (n = 5). Data are means ± SD.

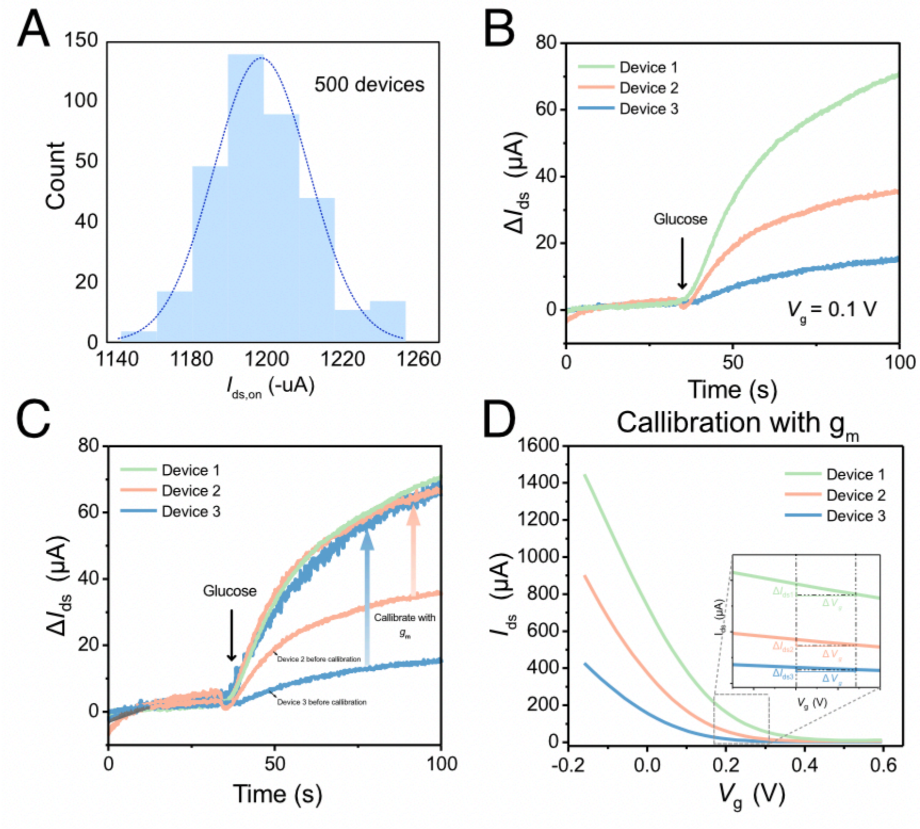

Fig. S9. Demonstration of self-calibration process with different devices. **(A)** Distribution of the on-state current for 500 OECT devices (with the same dimension). **(B)** The current response of three different OECT glucose sensors upon increasing the glucose concentration to 10 mM. Herein OECT devices with significant variation in performance were chosen to demonstrate the function of calibration. **(C)** The calibrated current response of OECT glucose sensors becomes identical. **(D)** The calibration is performed with their specific transfer curves and normalizing the associated G_m.

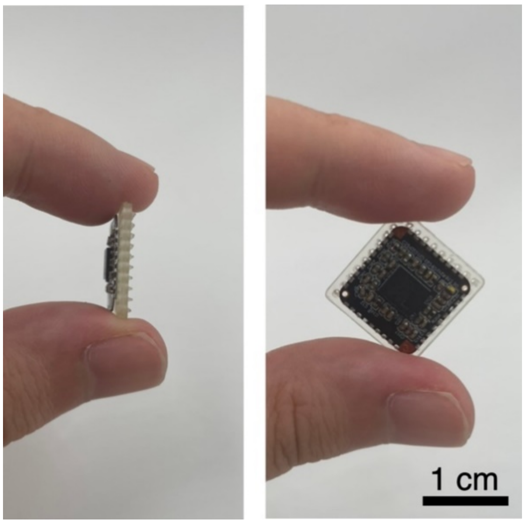

Fig. S10. Optical images of the prototype of OECT-CGM. The overall dimensions of the OECT-CGM are 15 mm in both length and width, with a thickness of 2.5 mm.

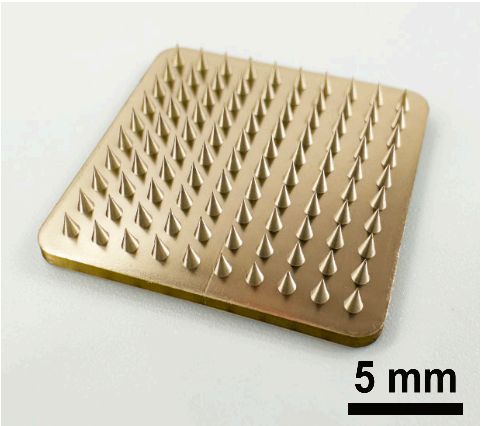

Fig. S11. The optical image of the microneedle array coated with Au. The microneedle has 10 × 10 arrays with sharpened tapered needles set at a 15-degree angle to ease skin penetration (*13*). A thin layer of Au was deposited on the surface to improve the biocompatibility.

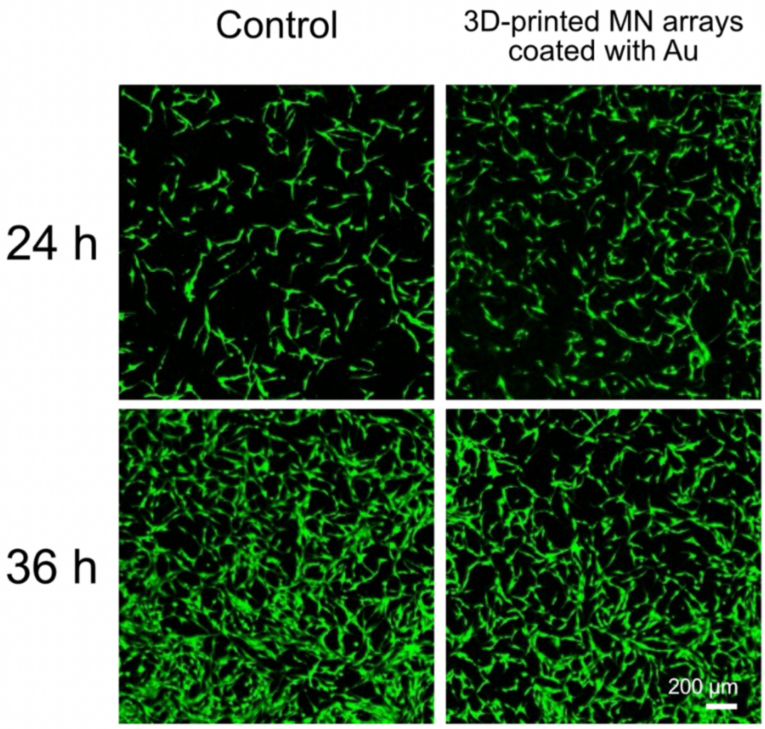

Fig. S12. Biocompatibility test of microneedle array. Cell viability of the microneedle coated with gold, compared with the control group, demonstrating a good biocompatibility of the gold-sputtered microneedles.

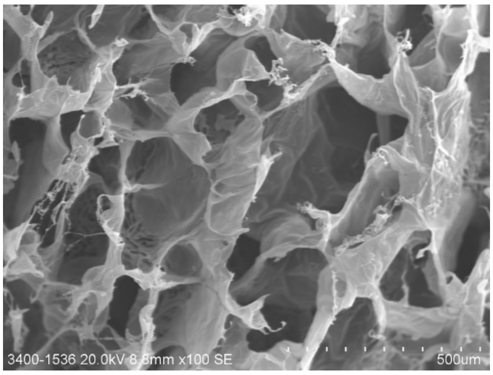

Fig. S13. Scanning electron microscope image (SEM) of the IPN hydrogel filler. The IPN hydrogel layer functions as a filler within hollow microneedles, designed to ease glucose diffusion from ISF to OECT. The SEM image illustrates the high porosity of the IPN hydrogel, which promotes glucose molecule diffusion.

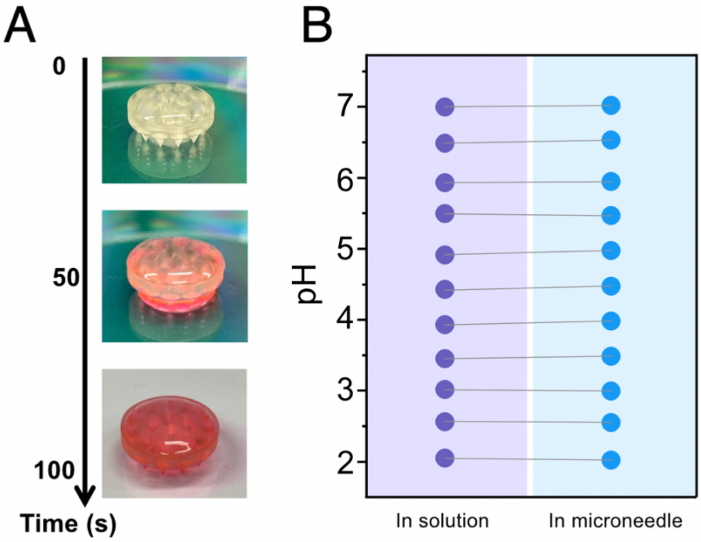

Fig. S14. Diffusive property characterization of the hydrogel. (A) Colorimetric test of diffusion in the hydrogel layer. Prior to gelation, the hydrogel was filled into a microneedle. The hydrogel layer had a thickness of approximately 300 μm. At t = 0 s, 50 μL of 0.1% methyl orange solution was dropped onto the tip of the microneedle, and photographs of the stained hydrogel were taken at t=50 s, 100 s, respectively, indicating the diffusive property of the hydrogel. (**B)** pH diffusion test in solution and in hydrogel-filled microneedle showed good consistency (pH values between 2 and 7), further confirming the diffusive property of the hydrogel, making it suitable to serve as the buffer layer between ISF and the sensor. The purple dots represent the pH values measured directly in the solution, while the blue dots represent the pH values measured in the hydrogel, after 200 s of stabilization following the addition of the solution.

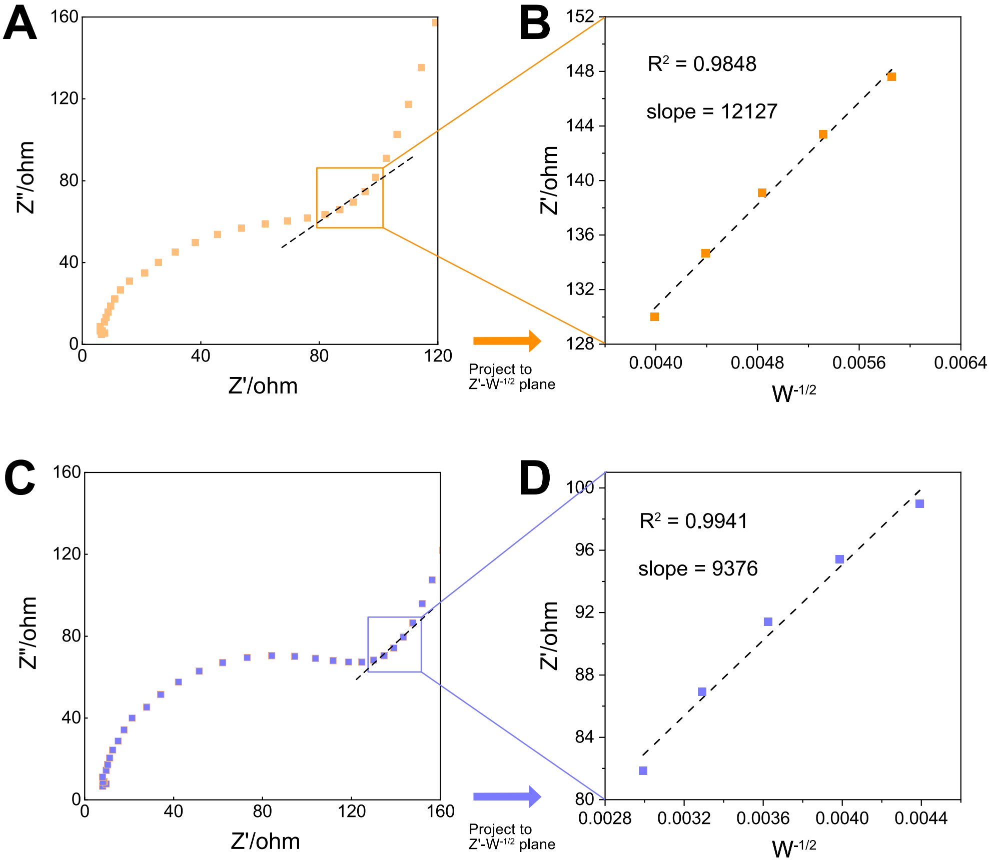

Fig. S15. Evaluating the glucose diffusion coefficient in the hydrogel with Nyquist plot and Warburg resistance. (A) Nyquist plots when using hydrogel-coated Au as the working electrode in an EC configurable. **(B)** Warburg element by analyzing the linear slope close to 45 degrees at low frequency. **(C)** Nyquist plots when using gold as the working electrode in an EC configurable. **(D)** Warburg element by analyzing the linear slope close to 45 degrees at low frequency.

Substituting the value into the formula (2), the diffusion coefficient of glucose in hydrogels and water can be estimated as:

$$D_{hydrogel} = \frac{8.314^2 \times 298.15^2}{2 \times 0.00785^2 \times 1^4 \times 96485^4 \times 0.001^2 \times 12127^2} = 3.90 \times 10^{-12}\ cm \cdot s^{-1}$$

$$D_{water} = \frac{8.314^2 \times 298.15^2}{2 \times 0.00785^2 \times 1^4 \times 96485^4 \times 0.001^2 \times 9376^2} = 6.54 \times 10^{-12}\ cm \cdot s^{-1}$$

To evaluate the diffusion efficiency of the hydrogel diffusion layer, we used EIS to measure the glucose diffusion coefficient (*74, 75*) by analyzing the Warburg impedance, a characteristic feature of EIS spectra at low frequencies (*76*). The diffusion of glucose molecules near the electrode can be described as Fick's second law of diffusion (*77*):

$$\frac{\partial c}{\partial t} = D\frac{\partial c^2}{\partial x^2} \quad (1)$$

Where C is glucose concentration, t is the time, x is the distance to the electrode, and D is the diffusion coefficient. The relationship between the D of glucose and the slope of the Warburg impedance can be expressed as (*78*):

$$D = \frac{R^2T^2}{2A^2n^4F^4C^2\sigma^2} \tag{2}$$

Where R is the ideal gas constant, T is the absolute temperature, A is the area of the electrode, n is the number of electrons transferred, F is Faraday's constant, C is the bulk glucose concentration, σ is the Warburg coefficient.

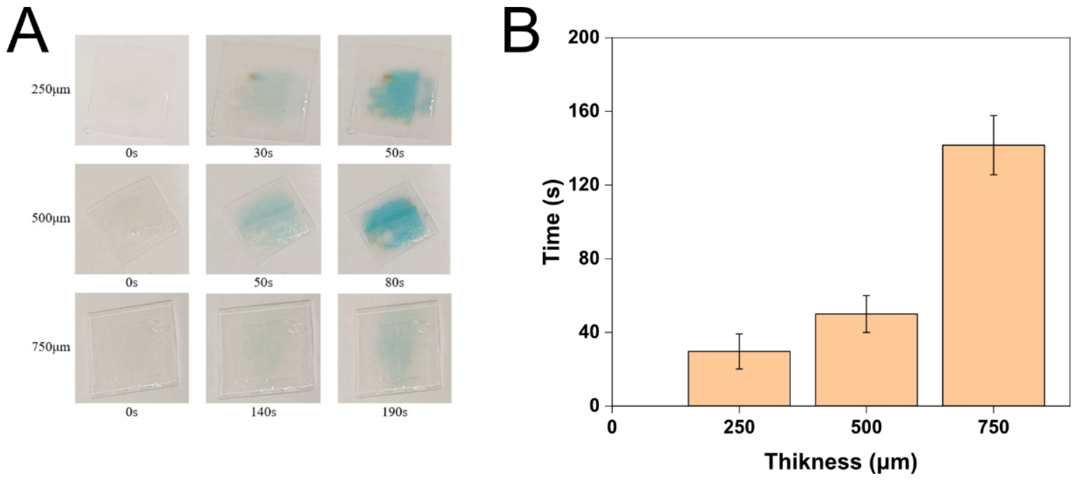

Fig. S16. Glucose diffusion time as a function of hydrogel film thickness. (A) Optical images of chromogenic diffusion experiments of hydrogel films of different thicknesses. **(B)** The X-axis represents the length of the needles (thickness of the hydrogels), varying at 250, 500, and 750 micrometers, while the Y-axis shows the diffusion time for a specific concentration of glucose. As the thickness of the hydrogel film increases, the time required for glucose to diffuse to the surface of the microneedle array correspondingly increases.

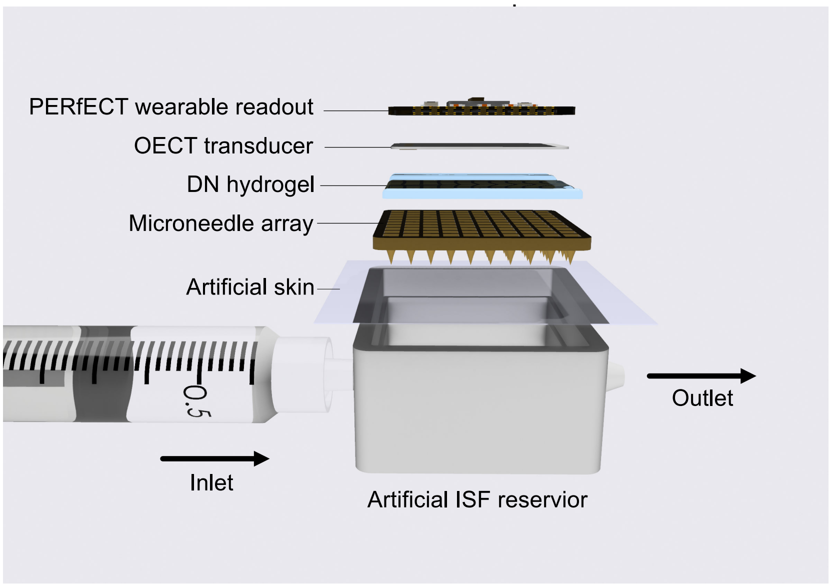

Fig. S17. *In-vitro* experiment setup of glucose monitoring in artificial ISF. The setup for testing the OECT-CGM *in-vitro* includes: i) an artificial ISF reservoir containing glucose solution on which the whole set of OECT-CGM can be placed stably; ii) a programmable syringe pump to inject glucose solutions of different concentrations into the reservoir at a constant speed; iii) an agarose-based artificial skin.

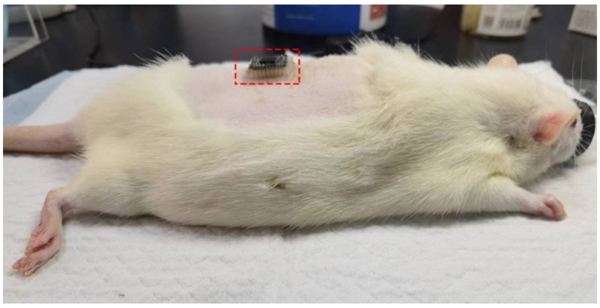

Fig. S18. ***In-vivo* continuous glucose measurement using the OECT-CGM system.** A real image of the *in-vivo* testing setup was employed to validate the performance of the OECT-CGM for real-time glucose concentration monitoring. The rat's dorsal area was carefully prepared by shaving it to facilitate the precise insertion of microneedles into the skin and to ensure optimal adhesion of the hydrogel. The highlighted area within the red dotted box represents the OECT-CGM device. In this configuration, glucose within the rat's tissue fluid naturally diffuses passively toward the OECT glucose sensor *via* the hydrogel-filled hollow microneedle. Subsequently, the sensor data is acquired by the miniaturized PERfECT readout system and wirelessly transmitted to a mobile phone using Bluetooth Low Energy (BLE) technology, where it is recorded and analyzed.

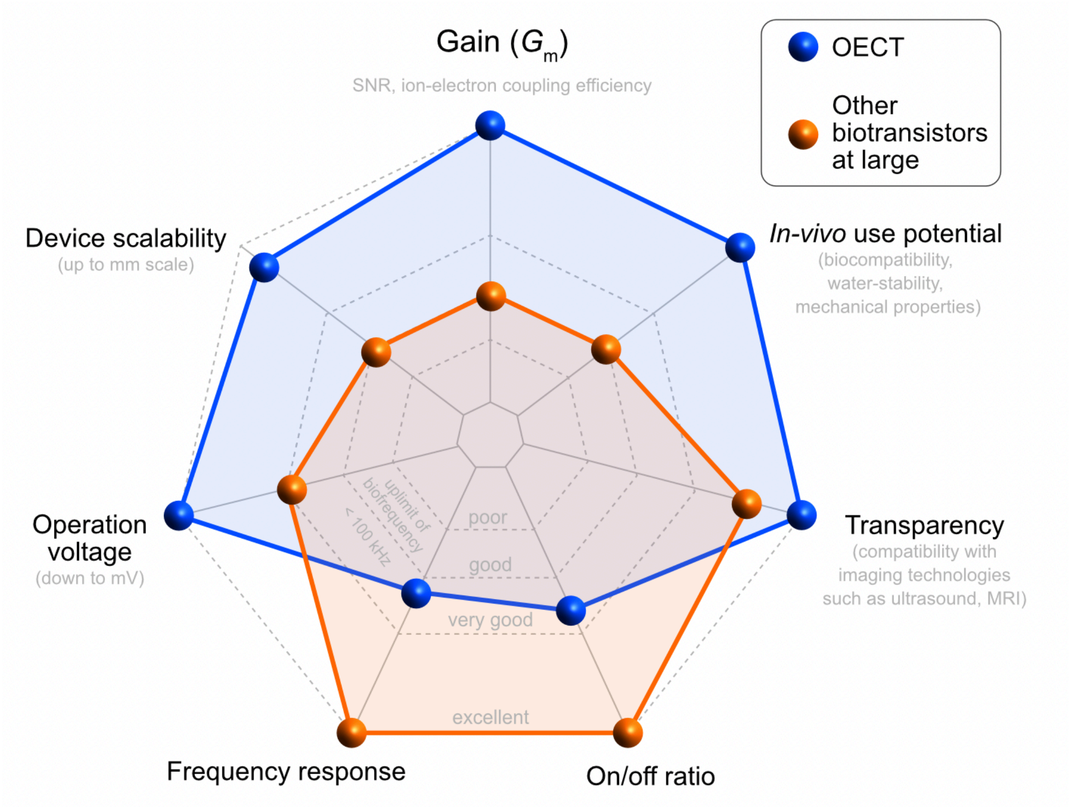

Fig. S19. Overall performance comparison between OECTs and other transistors. Overall comparison of OECT with other biotransistors (such as OFET), demonstrating its competitiveness to advance the current EC sensors for customizable and high-quality wearable biosensing applications.

Movie S1. The robust adhesion of OECT-CGM with skin.
